# B Cells Negatively Regulate the Establishment of CD49b^+^T-bet^+^ Resting Memory T Helper Cells in the Bone Marrow

**DOI:** 10.3389/fimmu.2016.00026

**Published:** 2016-02-02

**Authors:** Shintaro Hojyo, Jana Sarkander, Christian Männe, Mathias Mursell, Asami Hanazawa, David Zimmel, Jinfang Zhu, William E. Paul, Simon Fillatreau, Max Löhning, Andreas Radbruch, Koji Tokoyoda

**Affiliations:** ^1^Deutsches Rheuma-Forschungszentrum Berlin, Leibniz Institute, Berlin, Germany; ^2^Experimental Immunology and Osteoarthritis Research, Department of Rheumatology and Clinical Immunology, Charité-Universitätsmedizin Berlin, Berlin, Germany; ^3^Laboratory of Immunology, National Institute of Allergy and Infectious Diseases, National Institutes of Health, Bethesda, MD, USA; ^4^INSERM U1151-CNRS UMR 8253, Institut Necker-Enfants Malades, Paris, France; ^5^Faculté de Médecine, Université Paris Descartes, Sorbonne Paris Cité, Paris, France; ^6^Assistance Publique-Hôpitaux de Paris, Hôpital Necker Enfants Malades, Paris, France

**Keywords:** resting memory, CD4 T helper cells, B cells, bone marrow, T-bet

## Abstract

During an immune reaction, some antigen-experienced CD4 T cells relocate from secondary lymphoid organs (SLOs) to the bone marrow (BM) in a CD49b-dependent manner and reside and rest there as professional memory CD4 T cells. However, it remains unclear how the precursors of BM memory CD4 T cells are generated in the SLOs. While several studies have so far shown that B cell depletion reduces the persistence of memory CD4 T cells in the spleen, we here show that B cell depletion enhances the establishment of memory CD4 T cells in the BM and that B cell transfer conversely suppresses it. Interestingly, the number of antigen-experienced CD4 T cells in the BM synchronizes the number of CD49b^+^T-bet^+^ antigen-experienced CD4 T cells in the spleen. CD49b^+^T-bet^+^ antigen-experienced CD4 T cells preferentially localize in the red pulp area of the spleen and the BM in a T-bet-independent manner. We suggest that B cells negatively control the generation of CD49b^+^T-bet^+^ precursors of resting memory CD4 T cells in the spleen and may play a role in bifurcation of activated effector and resting memory CD4 T cell lineages.

## Introduction

One of the greatest characteristics of the immune system is to memorize previously encountered antigens and mount rapid recall responses. It is well established that CD4 T cells play an essential role in regulating the generation of high-affinity memory B cells and long-lived plasma cells (1–3) as well as the maintenance and expansion of memory CD8 T cells during secondary immune responses (4–7). However, the potential role and whereabouts of memory CD4 T cells were debated in the field for a long time. Memory CD4 T cells are generated from antigen-experienced CD4 T cells during the down-sizing of an immune reaction and are maintained in the absence of antigen by survival signals and homeostatic proliferation (8–11). While the bone marrow (BM) has been known to host long-lived plasma cells (12, 13), memory CD4 T cells were thought to constantly circulate throughout the body. Recently, we have uncovered that murine BM is a home of resting memory CD4 T cells (14). Some activated CD4 T cells migrate into the BM in a CD69- and CD49b (integrin α2)-dependent manner and reside and rest as memory CD4 T cells in the survival niches composed of IL-7^+^collagen-XI^+^ stromal cells (14–17). Interestingly, the repertoire of memory CD4 T cells in human BM is also significantly enriched for systemic pathogens compared to blood memory T cells (18, 19). While the vast majority of activated CD4 T cells undergo apoptosis after clearance of the antigen, it is still poorly understood which subpopulation in antigen-specific activated CD4 T cells has the potential to develop memory Th cells in the BM.

B cells are considered as potent antigen-presenting cells nearly as effective as dendritic cells (DCs) (20). While DCs play an essential role for priming naive CD4 T cells in the periarterial lymphatic sheaths (PALS) of spleen, B cells contribute to the generation of follicular helper T (Tfh) cells (21). Several reports provided direct evidence of the requirement of B cells for the establishment of memory CD4 T cells in the spleen, comparing the expansion and maintenance of antigen-specific CD4 T cells from B cell-depleted or -deficient and control mice (22–26). In case of a lymphocytic choriomeningitis virus (LCMV) infection model, B cell-deficient mice rapidly lost memory CD4 T cells in the spleen during the contraction phase, despite normal expansion after acute infection (26). Thus, B cells are important for the formation of splenic memory CD4 T cells in vaccination and infection models. However, all these reports focused on memory CD4 T cells in the spleen, but not in the BM.

In this study, we investigated whether B cells are involved in the establishment of resting CD4 T cell memory in the BM. We examined the accumulation of antigen-specific activated CD4 T cells in the BM of B cell-depleted or B cell-deficient mice in the early phase of an immune reaction. We here show that B cells make a negative impact on the accumulation of CD49b^+^T-bet^+^ resting memory CD4 T cells in the BM, suggesting that B cells contribute to a quantitative balance of the commitment to effector Tfh cell and BM resting memory CD4 T cell lineage.

## Materials and Methods

### Mice

Lymphocytic choriomeningitis virus–TCR tg [SMARTA (27)], *Tbx21*-KO (28), JHT (29), T-bet-ZsGreen reporter (30), or *Rag1*-KO mice were used. In all experiments, the mice were used at 6–16 weeks of age and were maintained under specific pathogen-free conditions. All mouse experiments were performed in accordance with the German law for animal protection and with permission from the local veterinary offices, and in compliance with the guidelines of the Institutional Animal Care and Use Committee. For immunizations, mice were injected intraperitoneally (i.p.) with LCMV GP_61–80_ peptide (synthesized by Genecust) plus lipopolysaccharide (LPS, O111:B4).

### Flow Cytometry and Cell Sorting

Single-cell suspensions were prepared from the spleen, BM, and blood of individual mice. The viability of cells was assessed by trypan blue exclusion. For cell staining, cells were stained for 15 min at 4°C with monoclonal antibodies against CD4 (RM4–5), CD19 (6D5), CD25 (PC61.5), CD44 (IM7), B220 (RA3-6B2), CD49b (HMa2), CD62L (MEL-14), NK1.1 (PK136), Thy1.1 (OX-7), CXCR4 (L276F12), CXCR5 (L138D7), CCR7 (4B12), and T-bet (4B10) and isotype controls. To exclude dead cells, we stained the cells with 1 μg/ml propidium iodide (Sigma). Intracellular staining for transcription factors was performed using Foxp3/transcription factor staining buffer kit (eBioscience) according to the manufacturer’s protocol.

### Real-Time PCR Analysis

The total RNA was extracted from splenocytes, and reverse-transcribed with High Capacity RNA-to-cDNA Kit (Life Technologies). Real-time PCR analysis was performed using the SYBR Green Master Mix (Life Technologies). Samples were normalized to the *Hprt* expression. *Cxcl12* primer sequences: forward 5′-AAA CCA GTC AGC CTG AGC TAC C-3′, reverse 5′-GGC TCT GGC GAT GTG GC-3′; *Hprt* primer sequences: forward 5′-TCC TCC TCA GAC CGC TTT T-3′, reverse 5′-CAT AAC CTG GTT CAT CAT CGC-3′.

### Cell Sorting and Adoptive Transfer

For positive sorting of splenic CD4 T cells, the Fab fragments of anti-CD4 antibody and Streptavidin MicroBeads (Miltenyi Biotec) were used. For negative sorting of splenic B cells, splenocytes were stained with FITC-conjugated anti-Mac1 (M1/70), anti-CD4 (GK1.5), and anti-CD8a (53–6.7) antibodies and then with anti-FITC and anti-Thy1.2 MicroBeads (Miltenyi Biotec) and were sorted by a magnetic cell separation system (MACS, Miltenyi Biotec). Approximately 0.5–1 × 10^6^ purified LCMV–TCR tg CD4 T cells were transferred intravenously (i.v.) into C57BL/6, JHT, or *Rag1*-KO mice, if any, with 1 × 10^7^ purified splenic B cells.

### B Cell Depletion

B cells were depleted by antibody-mediated antigen-receptor cross-linking, as previously described (31). Mice were injected i.p. with either 200 μg of anti-IgD (11.26c) or isotype control followed by injection of 200 μg of mouse anti-rat IgG antibody (MAR18.5). The efficiency of B cell depletion was examined by flow cytometric analysis using anti-B220 (RA3-6B2) and anti-CD19 (6D5) antibodies.

### Immunofluorescent Staining and Confocal Microscopy

For immunofluorescence staining, samples were fixed in 4% paraformaldehyde and equilibrated in 30% sucrose. Cryostat sections of adult spleen were stained with monoclonal antibodies against Thy1.1 (HIS51), B220 (RA3-6B2), and CD4 (RM4–5). All histological analyses were carried out with a confocal laser microscope (LSM710, Carl Zeiss).

### Statistical Analyses

All statistical analyses were performed using two-tailed Student’s *t*-test.

## Results

### B Cell Depletion Enhances the Accumulation of Antigen-Experienced CD4 T Cells in the BM

**Figure 1:**
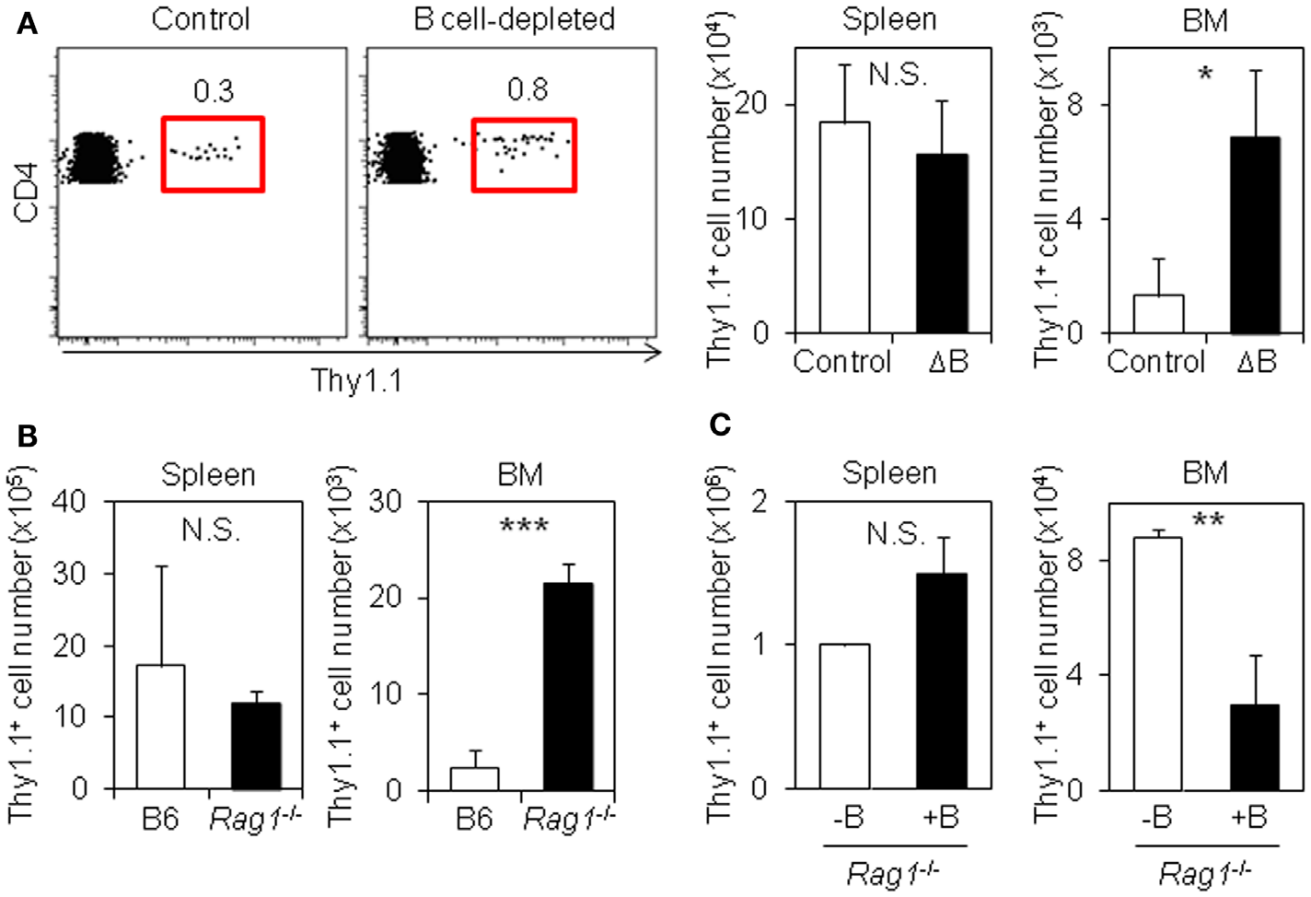
**B cells suppress the accumulation of antigen-specific CD4 T cells in the BM**. **(A)** B cell depletion promotes the accumulation of antigen-specific CD4 T cells in the BM. C57BL/6 mice were treated with isotype-matched control antibody or anti-IgD antibody followed by injection of anti-rat IgG antibody. Two days later, purified Thy1.1^+^ LCMV–TCR CD4 T cells were transferred into the antibody-treated mice, and then immunized with LCMV GP_61–80_ plus LPS. On day 6, the Thy1.1^+^ CD4 T cells in the spleen and BM were analyzed by flow cytometry and enumerated. Gating plots show the Thy1.1^+^ cell population in CD4^+^B220^−^NK1.1^−^PI^−^ cells of the BM. Bar charts represent the cell numbers. Data represent the mean ± SD; *p < 0.05; *N* = 4. **(B)** Accumulation of antigen-specific CD4 T cells in the BM is also promoted in *Rag1*-KO host mice. Purified Thy1.1^+^ LCMV–TCR CD4 T cells were transferred into C57BL/6 or *Rag1*-KO mice followed by immunization with LCMV GP_61–80_ plus LPS. On day 4, the Thy1.1^+^ CD4 T cells in the spleen and BM were analyzed by flow cytometry and enumerated. Bar charts represent the cell numbers. Data represent the mean ± SD; ***p < 0.001; *N* = 4. **(C)** B cell-cotransfer suppresses the accumulation of antigen-specific CD4 T cells into the BM. Purified Thy1.1^+^ LCMV–TCR CD4 T cells were transferred into *Rag1*-KO mice with or without B cells followed by immunization with LCMV GP_61–80_ plus LPS. On day 6, the Thy1.1^+^ CD4 T cells in the spleen and BM were analyzed by flow cytometry and enumerated. Bar charts represent the cell numbers. Data represent the mean ± SD; **p < 0.01; *N* = 4.

B cell depletion inhibits the generation of memory CD4 T cells in the spleen, while it does not affect the expansion of CD4 T cells (22–26). To examine the effect of B cell depletion on the generation of resting memory CD4 T cells in the BM, we transferred LCMV–TCR tg CD4 T cells into mice pretreated with anti-IgD or isotype control followed by treatment of anti-rat IgG. The depletion protocol reduced more than 80% of B220^hi^CD19^+^ mature B cells in the spleen and mesenteric lymph nodes (mLN), and around 70% in BM, but did not affect BM B cell precursors, including pro-B cells, which may compete for IL-7-expressing stromal niches with memory CD4 T cells (Figure S1 in Supplementary Material) (32, 33). Next, we immunized mice with LCMV peptide. Although LCMV-specific CD4 T cells in the spleen of B cell-depleted and control mice expanded similarly, there were more CD4 T cells in the BM of B cell-depleted mice compared to the BM of B cell-sufficient mice (Figure 1A). A similar result was obtained in the BM from *Rag1*^−/−^ host mice that had received antigen-specific CD4 T cells (Figure 1B). By contrast, when wild-type splenic polyclonal B cells were cotransferred with LCMV-specific CD4 T cells into *Rag1*^−/−^ mice followed by immunization with LCMV peptide (Figure S2A in Supplementary Material), the accumulation of antigen-specific CD4 T cells in the BM was dramatically decreased, despite normal expansion in the spleen (Figure 1C). Furthermore, in B cell-deficient JHT mice transferred with LCMV–TCR tg CD4 T cells, despite smaller expansion in the spleen compared to C57BL/6 host mice, the equivalent number of antigen-specific CD4 T cells was detected in the BM, involving in an increased ratio of antigen-specific CD4 T cells in the BM compared to the spleen (Figure S2B in Supplementary Material). Collectively, these results indicate that B cells suppress the accumulation of antigen-experienced CD4 T cells in the BM.

### B Cell Depletion Enhances the Induction of CD49b^+^T-bet^+^ Antigen-Specific CD4 T Cells

**Figure 2:**
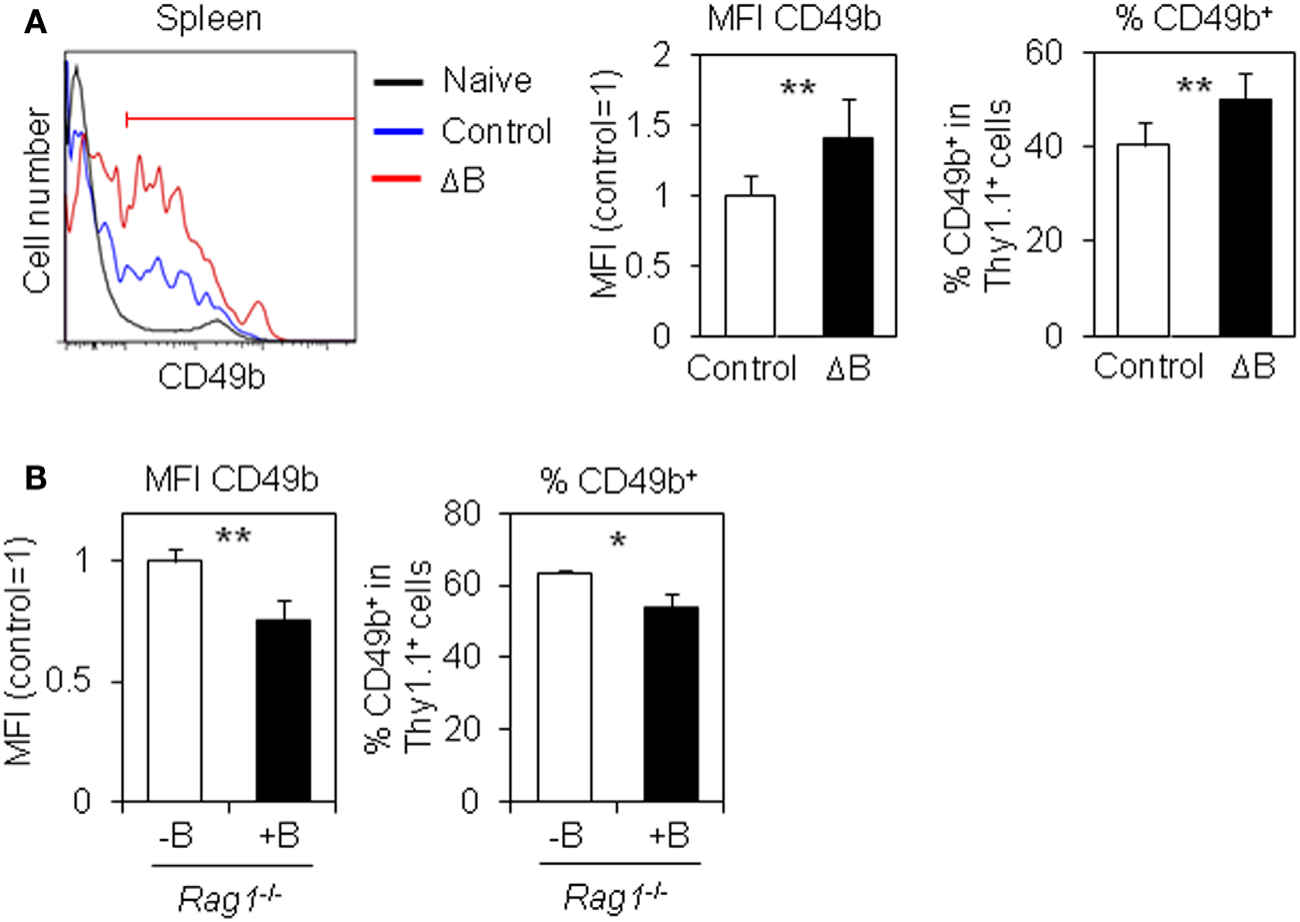
**CD49^+^ antigen-specific CD4 T cells are increased by B cell-depletion**. **(A)** CD49b^+^ antigen-specific CD4 T cells are increased in the spleen of B cell-depleted mice. Purified Thy1.1^+^ LCMV–TCR CD4 T cells were transferred into B cell-depleted or control C57BL/6 mice followed by immunization with LCMV GP_61–80_ plus LPS. On day 6, the CD49b^+^ population in the spleen was analyzed by flow cytometry. Histograms show CD49b expression in CD4^+^Thy1.1^+^B220^−^NK1.1^−^PI^−^ cells and CD4^+^Thy1.1^−^B220^−^NK1.1^−^PI^−^ (naive) cells in the spleen. Gated line in the histogram represents a CD49b^+^ population. Bar charts represent the relative ratio of mean fluorescent intensity (MFI) of CD49b (left) and CD49b^+^ population (right) in CD4^+^Thy1.1^+^B220^−^NK1.1^−^PI^−^ cells in the spleen. Data represent the mean ± SD; **p < 0.01; *N* = 7. **(B)** CD49b^+^ antigen-specific CD4 T cells are decreased in the spleen by B cell cotransfer. A CD49b^+^ population in the spleen of mice described in Figure 1C was analyzed by flow cytometry. Bar charts represent the relative ratio of MFI of CD49b (left) and CD49b^+^ population (right) in CD4^+^Thy1.1^+^B220^−^NK1.1^−^PI^−^ cells in the spleen. Data represent the mean ± SD; *p < 0.05, **p < 0.01; *N* = 3.

How does B cell depletion affect antigen-specific CD4 T cells in the spleen? CD49b^+^ antigen-specific activated CD4 T cells in the spleen preferentially migrate into the BM (15). We speculated that B cell depletion affects the expression of CD49b in antigen-specific activated CD4 T cells in the spleen. Notably, splenic antigen-specific CD4 T cells in B cell-depleted mice expressed significantly more CD49b compared to control mice (Figure 2A) and conversely the CD4 T cells in B cell-transferred *Rag1*^−/−^ mice decreased its expression (Figure 2B), suggesting that B cells negatively regulate the expression of CD49b and consequently reduce the accumulation of antigen-specific CD4 T cells in the BM.

**Figure 3:**
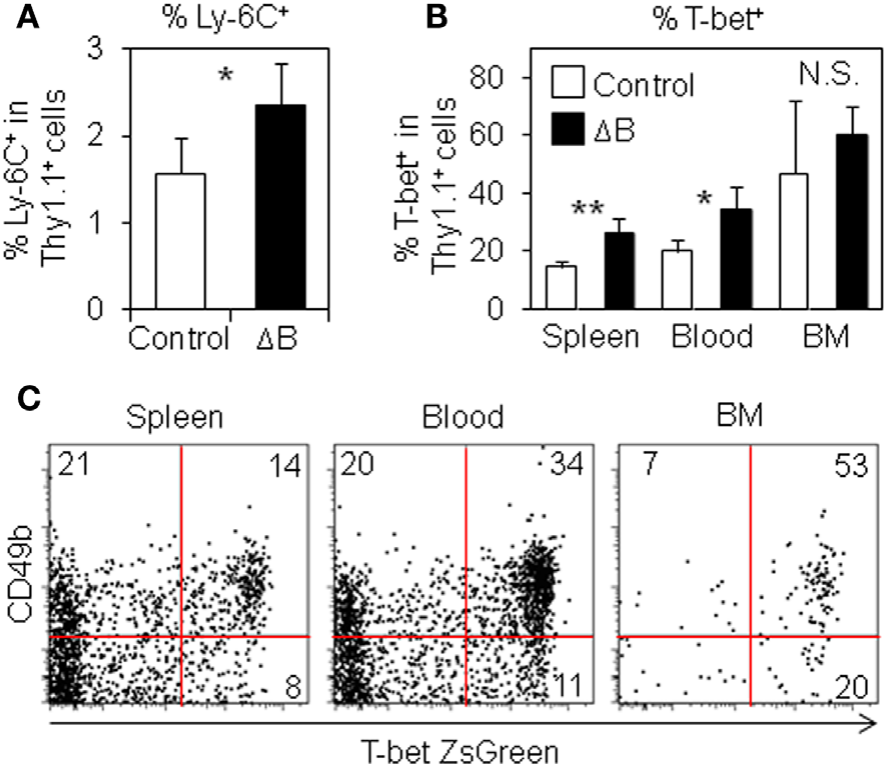
**Ly-6C^+^ and T-bet^+^ antigen-specific CD4 T cells are increased by B cell-depletion**. **(A)** Ly-6C^+^ antigen-specific CD4 T cells are increased in the spleen from B cell-depleted mice. Purified Thy1.1^+^ T-bet-ZsGreen reporter LCMV–TCR CD4 T cells were transferred into B cell-depleted or control C57BL/6 mice followed by immunization with LCMV GP_61–80_ plus LPS. On day 6, Ly-6C^+^ population in the spleen was analyzed by flow cytometry. Bar chart represents the Ly-6C^+^ population in CD4^+^Thy1.1^+^B220^−^NK1.1^−^PI^−^ cells in the spleen. Data represent the mean ± SD; *p < 0.05; *N* = 4. **(B)** T-bet^+^ antigen-specific CD4 T cells are increased in the spleen and blood from B cell-depleted mice. In **(A)**, T-bet^+^ (ZsGreen^+^) population in the spleen, blood, and BM was analyzed by flow cytometry. Bar chart represents T-bet^+^ (ZsGreen^+^) population in CD4^+^Thy1.1^+^B220^−^NK1.1^−^PI^−^ cells in the spleen, blood, and BM. Data represent the mean ± SD; *p < 0.05, **p < 0.01; *N* = 4. **(C)** CD49b^+^T-bet^+^ antigen-specific CD4 T cells are detected at the highest percentage in the BM. Purified Thy1.1^+^ T-bet-ZsGreen reporter LCMV–TCR CD4 T cells were transferred into C57BL/6 mice followed by immunization with LCMV GP_61–80_ plus LPS. On day 6, CD49b^+^T-bet^+^ (ZsGreen^+^) population in CD4^+^Thy1.1^+^B220^−^NK1.1^−^PI^−^ cells in the spleen, blood, and BM was analyzed by flow cytometry.

Misumi and Whitmire have reported that B cell depletion affects the expression of Ly-6C and T-bet in antigen-specific CD4 T cells (23). Therefore, in order to examine the expression of Ly-6C and T-bet in antigen-specific CD4 T cells, we used anti-Ly-6C antibodies and T-bet ZsGreen BAC tg mice that express ZsGreen fluorescence protein under control of *Tbx21* regulatory elements (30). Ly-6C^+^ cells were slightly but significantly increased in splenic antigen-specific CD4 T cells from B cell-depleted mice (Figure 3A). T-bet^+^ antigen-specific CD4 T cells were significantly increased in the spleen and blood after B cell depletion, while the population in the BM was likely saturated (Figure 3B). We also detected three times more T-bet^+^ cells among antigen-specific CD4 T cells in the spleen of B cell-depleted mice using a T-bet-specific antibody (Figure S3 in Supplementary Material). Intriguingly, CD49b^+^T-bet^+^ antigen-specific CD4 T cells in immunized host mice were detected at the lowest percentage in the spleen (14%), at the midst in the blood (34%), and at the highest in the BM (53%) (Figure 3C), suggesting that CD49b^+^T-bet^+^ antigen-specific CD4 T cells selectively migrate from the spleen into the BM *via* blood. These results argue that CD49b^+^T-bet^+^ antigen-specific CD4 T cells are the potential precursors of BM resting memory CD4 T cells.

### T-bet^+^ Antigen-Specific CD4 T Cells Preferentially Localize in Red Pulp of Spleen

**Figure 4:**
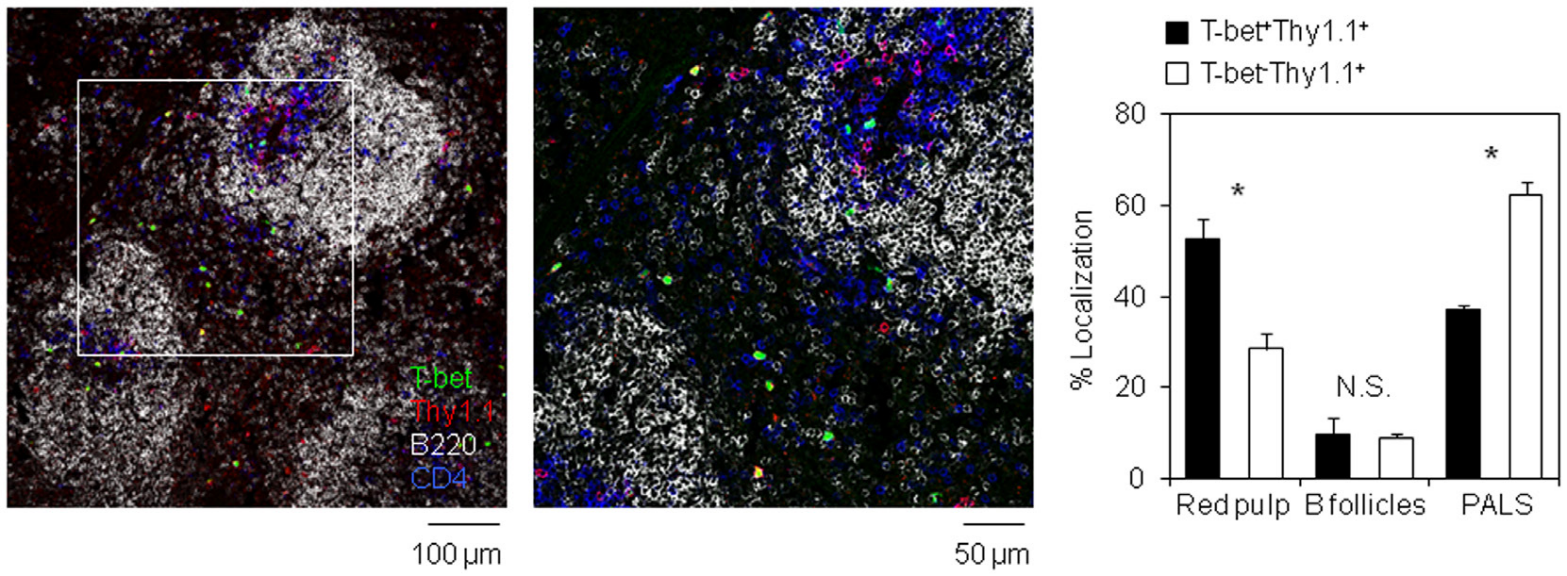
**T-bet-expressing antigen-specific CD4 T cells preferentially egress from white pulp into red pulp**. Localization of T-bet^+^ (ZsGreen^+^) Thy1.1^+^ CD4 T cells in the spleen section. Frozen section prepared from the spleen of host mouse in Figure 3C was stained with anti-Thy1.1 (red), anti-B220 (gray), and anti-CD4 (blue) antibodies. Bar chart represents the localization of T-bet^+^Thy1.1^+^ and T-bet^−^Thy1.1^+^ cells in each area of the spleen. Data represent the mean ± SD. *p < 0.05.

The precursors of long-lived plasma cells egress from splenic B cell follicles toward the BM *via* splenic red pulp and blood (34). To examine the localization of T-bet^+^ antigen-specific CD4 T cells in the spleen, we performed a histological analysis and assessed the localization of T-bet^+^ and T-bet^−^ antigen-specific CD4 T cells (Figure 4). On day 6 after immunization, while most of T-bet^−^ antigen-specific CD4 T cells remained in the white pulp, including B cell follicles and PALS, T-bet^+^ cells significantly stayed in the red pulp. These data suggest that T-bet^+^ resting memory CD4 T cell precursors preferentially localize in splenic red pulp and blood. It is well known that the precursors of long-lived plasma cells migrate into the BM in a CXCL12-dependent manner (33, 35). To examine the involvement of some chemokines in the localization of the resting memory CD4 T cell precursors, the expression of *Cxcl12* mRNA in the spleen tissue and CXCR4, CXCR5, and CCR7 proteins on antigen-specific CD4 T cells in the spleen and BM from B-cell depleted and control mice were analyzed. However, their expression profiles were not influenced by B cell depletion (Figure S4 in Supplementary Material).

### T-bet Is Not Required for the Accumulation of Antigen-Specific CD4 T Cells in the BM

**Figure 5:**
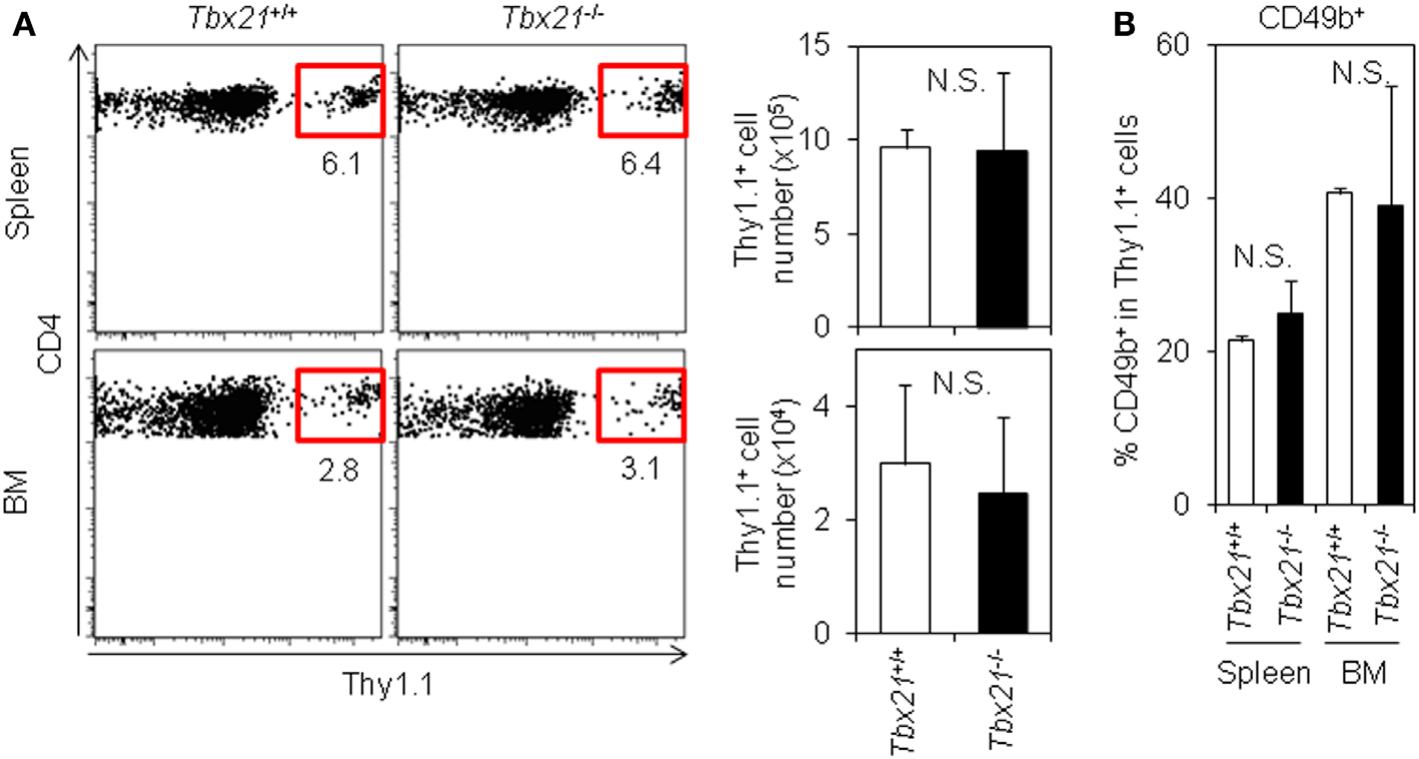
**T-bet is not required for the accumulation of antigen-specific CD4 T cells in the BM**. **(A)** T-bet-deficient CD4 T cells normally accumulate in the BM. Purified *Tbx21*^+^*^/^*^+^ or *Tbx21*^−/−^ Thy1.1^+^ LCMV–TCR CD4 T cells were transferred into C57BL/6 mice followed by immunization with LCMV GP_61–80_ plus LPS. On day 6, the Thy1.1^+^ CD4 T cells in the spleen and BM were analyzed by flow cytometry and enumerated. Gating plots show a Thy1.1^+^ population in CD4^+^B220^−^PI^−^ cells in the spleen and BM. Bar charts represent the cell numbers. Data represent the mean ± SD; *N* = 3. **(B)** T-bet-deficiency does not alter surface CD49b expression. In **(A)**, CD49b^+^ population in antigen-specific CD4 T cells was analyzed by flow cytometry. Bar chart represents CD49b^+^ population in CD4^+^Thy1.1^+^B220^−^PI^−^ cells in the spleen and BM. Data represent the mean ± SD; *N* = 3.

Finally, to examine the physiological impact of T-bet on the accumulation of antigen-specific CD4 T cells in the BM, we compared the accumulation of T-bet^+/+^ or T-bet^−/−^ LCMV–TCR tg CD4 T cells in the BM. On day 6 after immunization, T-bet-deficient CD4 T cells accumulated in the BM equally as well as T-bet-sufficient cells (Figure 5A). Furthermore, T-bet deficiency did not affect surface CD49b expression (Figure 5B). Consistent with these observations, when we compared the number of CD44^hi^ memory-phenotype CD4 T cells in T-bet-sufficient and -deficient backgrounds, both groups showed similar numbers of CD44^hi^ CD4 T cells in the spleen and BM (Figure S5 in Supplementary Material). Taken together, T-bet is dispensable for the expression of CD49b to facilitate the accumulation of antigen-specific CD4 T cells in the BM.

## Discussion

B cells have been described as a positive regulator to generate CD4 T cell memory in the spleen (22–26). However, we here showed that the depletion or deficiency of B cells actually promotes the accumulation of antigen-specific activated CD4 T cells in the BM, and in contrast the pretransfer of B cells suppresses their accumulation, suggesting that B cells are a negative regulator to generate CD4 T cell memory in the BM. We also found that B cell depletion facilitates the upregulation of the expression of CD49b, a homing receptor of CD4 T cells to the BM, and T-bet, the lineage-specifying transcription factor of Th1 cell differentiation, in the activated CD4 T cells in the spleen. Moreover, the T-bet^+^ cell population preferentially localizes in the red pulp of the spleen and in the BM during an immune reaction. We suggest that CD49b^+^T-bet^+^ antigen-specific CD4 T cells in the spleen are the precursors of BM resting memory CD4 T cells.

We demonstrated that B cells negatively control the expression of T-bet and CD49b in antigen-experienced CD4 T cells in the spleen. It remains unclear at the molecular level how B cells downregulate the expression of these molecules and whether a T-bet-inducing signal is directly involved in CD49b expression in activated CD4 T cells. Intriguingly, a loss of T-bet did not alter the expression level of CD49b in activated CD4 T cells and hardly affected their accumulation in the BM. The result suggests that T-bet itself plays a dispensable role in the migration of activated CD4 T cells. Rather, the upstream signaling pathway (T-bet-inducing signal) might be critical in this process in parallel with the control of CD49b expression. Naive T cells do not express T-bet and CD49b. Once they are stimulated *via* the TCR, while T-bet expression is induced *via* IFNγR and IL-12R signaling pathways (28, 30, 36, 37), CD49b expression is induced as well as VLA-2 on Th1 cells *in vitro* in an IFNγ- and IL-12-independent manner (38). Hence, opposite to the upregulation of T-bet and CD49b by DCs, B cell-mediated signals may commonly downregulate the expression of these molecules. This notion is supported by recent findings (39, 40). They have described that ICOS costimulation *via* B cells is crucial to maintain the Tfh phenotype through the downregulation of transcription factor KLF2 and that the blockade of ICOS ligand on B cells increases *Tbx21* transcripts in antigen-specific CD4 T cells. In a KLF2-transduced HUVEC cell line, CD49b is upregulated compared to a control cell line at the transcriptional level (41). It remains to be further clarified whether B cells suppress T-bet and CD49b in activated CD4 T cells in a KLF2-dependent manner.

Our data suggest that DCs may activate CD4 T cells and license them to differentiate into resting memory cells in the BM during primary immune response, while some activated CD4 T cells contact bystander B cells as follow-up antigen-presenting cells through cognate interaction and further differentiate into effector Tfh cells (42–45). Thus, additional activation by B cells following DCs may induce the differentiation of effector Tfh cell lineage (21), suppressing the differentiation program for a BM resting memory cell lineage. Resting memory CD4 T cells in the BM are more functional *in vitro* and *in vivo* compared to spleen-resident memory cells (14). Adoptively transferred memory CD4 T cells from the BM can efficiently help B cells to produce high-affinity antibodies, suggesting that BM memory CD4 T cells can differentiate into Tfh cells during recall response. We have previously shown that a persistent antigen with adjuvants of oil and aluminum hydroxide enhances the expansion of antigen-specific CD4 T cells in the secondary lymphoid organs (SLOs), but not the BM (46). BM resting memory CD4 T cells are unaffected by persistence of antigen (46), while splenic Tfh cells, probably also memory Tfh cells, are sustained by persistence of antigen (47). Thus, the ratio of splenic Tfh cells and BM resting memory cells is markedly affected by antigen persistence. We suggest that the length of antigen persistence may define the quantitative balance of effector and resting memory, i.e., whether the CD4 T cells should contribute to the long-lasting reaction to exclude the persistent antigen or store their ability for recall response.

## Author Contributions

SH, JS, and KT designed the research; SH, JS, CM, MM, and DZ performed the research; AH, JZ, WP, SF, and ML contributed new reagents/analytic tools; and SH, ML, AR, and KT wrote the manuscript.

## Conflict of Interest Statement

The authors declare that the research was conducted in the absence of any commercial or financial relationships that could be construed as a potential conflict of interest.

## Abbreviations

BM, bone marrow; DC, dendritic cell; LCMV, lymphocytic choriomeningitis virus; PALS, periarterial lymphatic sheaths; SLO, secondary lymphoid organ; Tfh, T follicular helper.

## References

[1] Crotty S Kersh EN Cannons J Schwartzberg PL Ahmed R. SAP is required for generating long-term humoral immunity. Nature (2003) 421:282–7. 10.1038/nature01318 12529646

[2] Francus T Francus Y Siskind GW. Memory T cells enhance the expression of high-avidity naive B cells. Cell Immunol (1991) 134:520–7. 10.1016/0008-8749(91)90323-4 2021976

[3] Gershon RK Paul WE Effect of thymus-derived lymphocytes on amount and affinity of anti-hapten antibody. J Immunol (1971) 106:872–4. 5102123

[4] Janssen EM Lemmens EE Wolfe T Christen U von Herrath MG Schoenberger SP. CD4+ T cells are required for secondary expansion and memory in CD8+ T lymphocytes. Nature (2003) 421:852–6. 10.1038/nature01441 12594515

[5] Shedlock DJ Shen H. Requirement for CD4 T cell help in generating functional CD8 T cell memory. Science (2003) 300:337–9. 10.1126/science.1082305 12690201

[6] Sun JC Bevan MJ. Defective CD8 T cell memory following acute infection without CD4 T cell help. Science (2003) 300:339–42. 10.1126/science.1083317 12690202 PMC2778341

[7] Sun JC Williams MA Bevan MJ. CD4+ T cells are required for the maintenance, not programming, of memory CD8+ T cells after acute infection. Nat Immunol (2004) 5:927–33. 10.1038/ni1105 15300249 PMC2776074

[8] Boyman O Letourneau S Krieg C Sprent J Homeostatic proliferation and survival of naive and memory T cells. Eur J Immunol (2009) 39:2088–94. 10.1002/eji.200939444 19637200

[9] Kassiotis G Garcia S Simpson E Stockinger B. Impairment of immunological memory in the absence of MHC despite survival of memory T cells. Nat Immunol (2002) 3:244–50. 10.1038/ni766 11836529

[10] Seddon B Tomlinson P Zamoyska R. Interleukin 7 and T cell receptor signals regulate homeostasis of CD4 memory cells. Nat Immunol (2003) 4:680–6. 10.1038/ni946 12808452

[11] Swain SL Hu H Huston G. Class II-independent generation of CD4 memory T cells from effectors. Science (1999) 286:1381–3. 10.1126/science.286.5443.1381 10558997

[12] Manz RA Thiel A Radbruch A Lifetime of plasma cells in the bone marrow. Nature (1997) 388:133–4. 10.1038/40540 9217150

[13] Slifka MK Antia R Whitmire JK Ahmed R. Humoral immunity due to long-lived plasma cells. Immunity (1998) 8:363–72. 10.1016/S1074-7613(00)80541-5 9529153

[14] Tokoyoda K Zehentmeier S Hegazy AN Albrecht I Grun JR Lohning M Professional memory CD4+ T lymphocytes preferentially reside and rest in the bone marrow. Immunity (2009) 30:721–30. 10.1016/j.immuni.2009.03.015 19427242

[15] Hanazawa A Hayashizaki K Shinoda K Yagita H Okumura K Lohning M CD49b-dependent establishment of T helper cell memory. Immunol Cell Biol (2013) 91:524–31. 10.1038/icb.2013.36 23897120

[16] Kassiotis G Gray D Kiafard Z Zwirner J Stockinger B. Functional specialization of memory Th cells revealed by expression of integrin CD49b. J Immunol (2006) 177:968–75. 10.4049/jimmunol.177.2.968 16818752

[17] Shinoda K Tokoyoda K Hanazawa A Hayashizaki K Zehentmeier S Hosokawa H Type II membrane protein CD69 regulates the formation of resting T-helper memory. Proc Natl Acad Sci U S A (2012) 109:7409–14. 10.1073/pnas.1118539109 22474373 PMC3358871

[18] Herndler-Brandstetter D Landgraf K Jenewein B Tzankov A Brunauer R Brunner S Human bone marrow hosts polyfunctional memory CD4+ and CD8+ T cells with close contact to IL-15-producing cells. J Immunol (2011) 186:6965–71. 10.4049/jimmunol.1100243 21562158

[19] Okhrimenko A Grun JR Westendorf K Fang Z Reinke S von Roth P Human memory T cells from the bone marrow are resting and maintain long-lasting systemic memory. Proc Natl Acad Sci U S A (2014) 111:9229–34. 10.1073/pnas.1318731111 24927527 PMC4078840

[20] Yuseff MI Pierobon P Reversat A Lennon-Dumenil AM. How B cells capture, process and present antigens: a crucial role for cell polarity. Nat Rev Immunol (2013) 13:475–86. 10.1038/nri3469 23797063

[21] Crotty S. T follicular helper cell differentiation, function, and roles in disease. Immunity (2014) 41:529–42. 10.1016/j.immuni.2014.10.004 25367570 PMC4223692

[22] Linton PJ Harbertson J Bradley LM. A critical role for B cells in the development of memory CD4 cells. J Immunol (2000) 165:5558–65. 10.4049/jimmunol.165.10.5558 11067910

[23] Misumi I Whitmire JK. B cell depletion curtails CD4+ T cell memory and reduces protection against disseminating virus infection. J Immunol (2014) 192:1597–608. 10.4049/jimmunol.1302661 24453250 PMC3925510

[24] Mollo SB Zajac AJ Harrington LE. Temporal requirements for B cells in the establishment of CD4 T cell memory. J Immunol (2013) 191:6052–9. 10.4049/jimmunol.1302033 24218454 PMC3866023

[25] van Essen D Dullforce P Brocker T Gray D. Cellular interactions involved in Th cell memory. J Immunol (2000) 165:3640–6. 10.4049/jimmunol.165.7.3640 11034367

[26] Whitmire JK Asano MS Kaech SM Sarkar S Hannum LG Shlomchik MJ Requirement of B cells for generating CD4+ T cell memory. J Immunol (2009) 182:1868–76. 10.4049/jimmunol.0802501 19201839 PMC2658628

[27] Oxenius A Bachmann MF Zinkernagel RM Hengartner H. Virus-specific MHC-class II-restricted TCR-transgenic mice: effects on humoral and cellular immune responses after viral infection. Eur J Immunol (1998) 28:390–400. 10.1002/(SICI)1521-4141(199801)28:01<390::AID-IMMU390>3.3.CO;2-F 9485218

[28] Szabo SJ Kim ST Costa GL Zhang X Fathman CG Glimcher LH. A novel transcription factor, T-bet, directs Th1 lineage commitment. Cell (2000) 100:655–69. 10.1016/S0092-8674(00)80702-3 10761931

[29] Gu H Zou YR Rajewsky K. Independent control of immunoglobulin switch recombination at individual switch regions evidenced through Cre-loxP-mediated gene targeting. Cell (1993) 73:1155–64. 10.1016/0092-8674(93)90644-6 8513499

[30] Zhu J Jankovic D Oler AJ Wei G Sharma S Hu G The transcription factor T-bet is induced by multiple pathways and prevents an endogenous Th2 cell program during Th1 cell responses. Immunity (2012) 37:660–73. 10.1016/j.immuni.2012.09.007 23041064 PMC3717271

[31] Tsubata T Murakami M Honjo T. Antigen-receptor cross-linking induces peritoneal B-cell apoptosis in normal but not autoimmunity-prone mice. Curr Biol (1994) 4:8–17. 10.1016/S0960-9822(00)00003-8 7922322

[32] Hardy RR Kincade PW Dorshkind K. The protean nature of cells in the B lymphocyte lineage. Immunity (2007) 26:703–14. 10.1016/j.immuni.2007.05.013 17582343

[33] Tokoyoda K Egawa T Sugiyama T Choi BI Nagasawa T. Cellular niches controlling B lymphocyte behavior within bone marrow during development. Immunity (2004) 20:707–18. 10.1016/j.immuni.2004.05.001 15189736

[34] Manz RA Arce S Cassese G Hauser AE Hiepe F Radbruch A. Humoral immunity and long-lived plasma cells. Curr Opin Immunol (2002) 14:517–21. 10.1016/S0952-7915(02)00356-4 12088688

[35] Hargreaves DC Hyman PL Lu TT Ngo VN Bidgol A Suzuki G A coordinated change in chemokine responsiveness guides plasma cell movements. J Exp Med (2001) 194:45–56. 10.1084/jem.194.1.45 11435471 PMC2193440

[36] Afkarian M Sedy JR Yang J Jacobson NG Cereb N Yang SY T-bet is a STAT1-induced regulator of IL-12R expression in naive CD4^+^ T cells. Nat Immunol (2002) 3:549–57. 10.1038/ni794 12006974

[37] Schulz EG Mariani L Radbruch A Hofer T. Sequential polarization and imprinting of type 1 T helper lymphocytes by interferon-gamma and interleukin-12. Immunity (2009) 30:673–83. 10.1016/j.immuni.2009.03.013 19409816

[38] Sasaki K Tsuji T Jinushi T Matsuzaki J Sato T Chamoto K Differential regulation of VLA-2 expression on Th1 and Th2 cells: a novel marker for the classification of Th subsets. Int Immunol (2003) 15:701–10. 10.1093/intimm/dxg066 12750354

[39] Lee JY Skon CN Lee YJ Oh S Taylor JJ Malhotra D The transcription factor KLF2 restrains CD4(+) T follicular helper cell differentiation. Immunity (2015) 42:252–64. 10.1016/j.immuni.2015.01.013 25692701 PMC4409658

[40] Weber JP Fuhrmann F Feist RK Lahmann A Al Baz MS Gentz LJ ICOS maintains the T follicular helper cell phenotype by down-regulating Kruppel-like factor 2. J Exp Med (2015) 212:217–33. 10.1084/jem.20141432 25646266 PMC4322049

[41] Parmar KM Larman HB Dai G Zhang Y Wang ET Moorthy SN Integration of flow-dependent endothelial phenotypes by Kruppel-like factor 2. J Clin Invest (2006) 116:49–58. 10.1172/JCI24787 16341264 PMC1307560

[42] Crotty S Johnston RJ Schoenberger SP. Effectors and memories: Bcl-6 and Blimp-1 in T and B lymphocyte differentiation. Nat Immunol (2010) 11:114–20. 10.1038/ni.1837 20084069 PMC2864556

[43] Hale JS Youngblood B Latner DR Mohammed AU Ye L Akondy RS Distinct memory CD4+ T cells with commitment to T follicular helper- and T helper 1-cell lineages are generated after acute viral infection. Immunity (2013) 38:805–17. 10.1016/j.immuni.2013.02.020 23583644 PMC3741679

[44] Marshall HD Chandele A Jung YW Meng H Poholek AC Parish IA Differential expression of Ly6C and T-bet distinguish effector and memory Th1 CD4(+) cell properties during viral infection. Immunity (2011) 35:633–46. 10.1016/j.immuni.2011.08.016 22018471 PMC3444169

[45] Pepper M Pagan AJ Igyarto BZ Taylor JJ Jenkins MK. Opposing signals from the Bcl6 transcription factor and the interleukin-2 receptor generate T helper 1 central and effector memory cells. Immunity (2011) 35:583–95. 10.1016/j.immuni.2011.09.009 22018468 PMC3208313

[46] Hanazawa A Lohning M Radbruch A Tokoyoda K. CD49b/CD69-dependent generation of resting t helper cell memory. Front Immunol (2013) 4:183. 10.3389/fimmu.2013.00183 23847623 PMC3706785

[47] Baumjohann D Preite S Reboldi A Ronchi F Ansel KM Lanzavecchia A Persistent antigen and germinal center B cells sustain T follicular helper cell responses and phenotype. Immunity (2013) 38:596–605. 10.1016/j.immuni.2012.11.020 23499493

