# Supplementary material for: B Cells Negatively Regulate the Establishment of CD49b+T-bet+ Resting Memory T Helper Cells in the Bone Marrow
Source: Front Immunol. 2016 Feb 2;7:26. doi: 10.3389/fimmu.2016.00026 (PMC4735404; doi:10.3389/fimmu.2016.00026)
Supplement: Supplementary file 1 [file Data_Sheet_1.PDF]

Supplementary Material

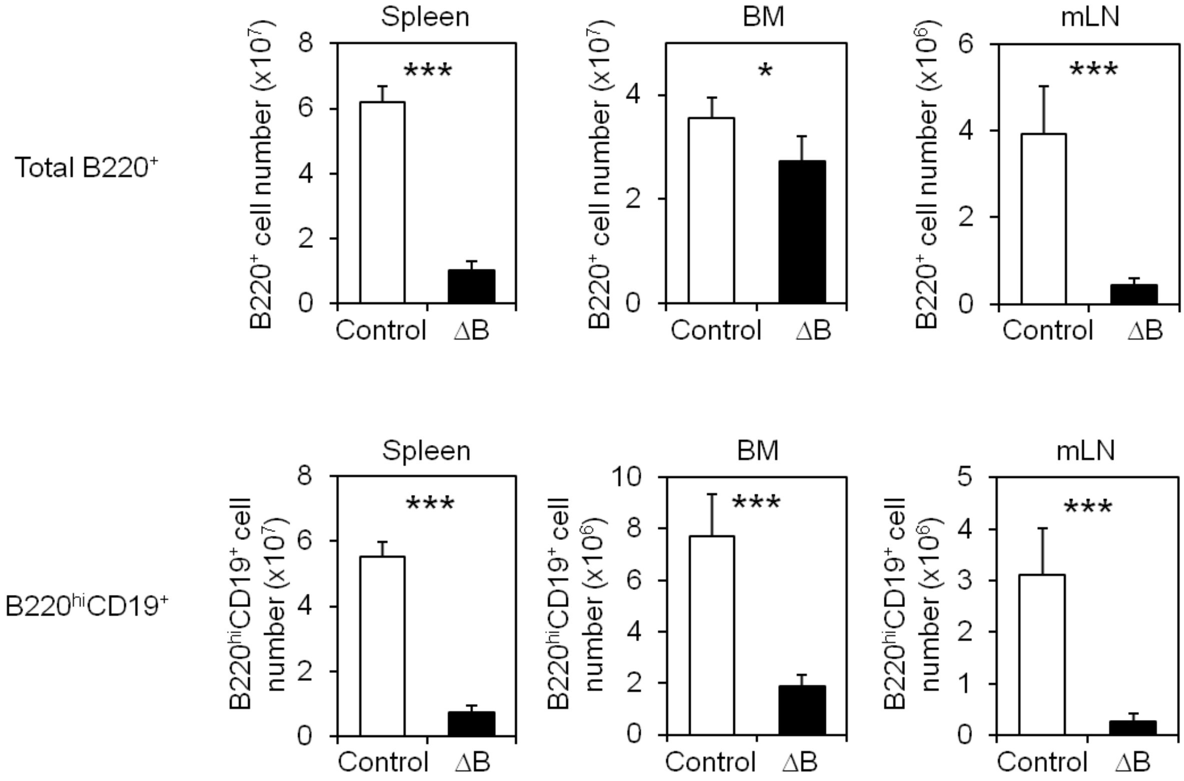

Figure S1 B cell-depletion by antibody-mediated antigen receptor cross-linking

Efficiency of B cell-depletion. C57BL/6 mice were treated with isotype-matched control or anti-IgD antibody followed by injection of anti-rat IgG antibody. Eight days later, B cells in the spleen, BM and mesenteric lymph nodes (mLN) were analyzed by flow cytometry and enumerated. Bar charts represent the $B220^{+}$ (upper panels) and $B220^{hi}CD19^{+}$ (lower panels) cell numbers. Data are representative of two independent experiments and represent the mean ± SD. * $p < 0.05$, *** $p < 0.001$. N=6.

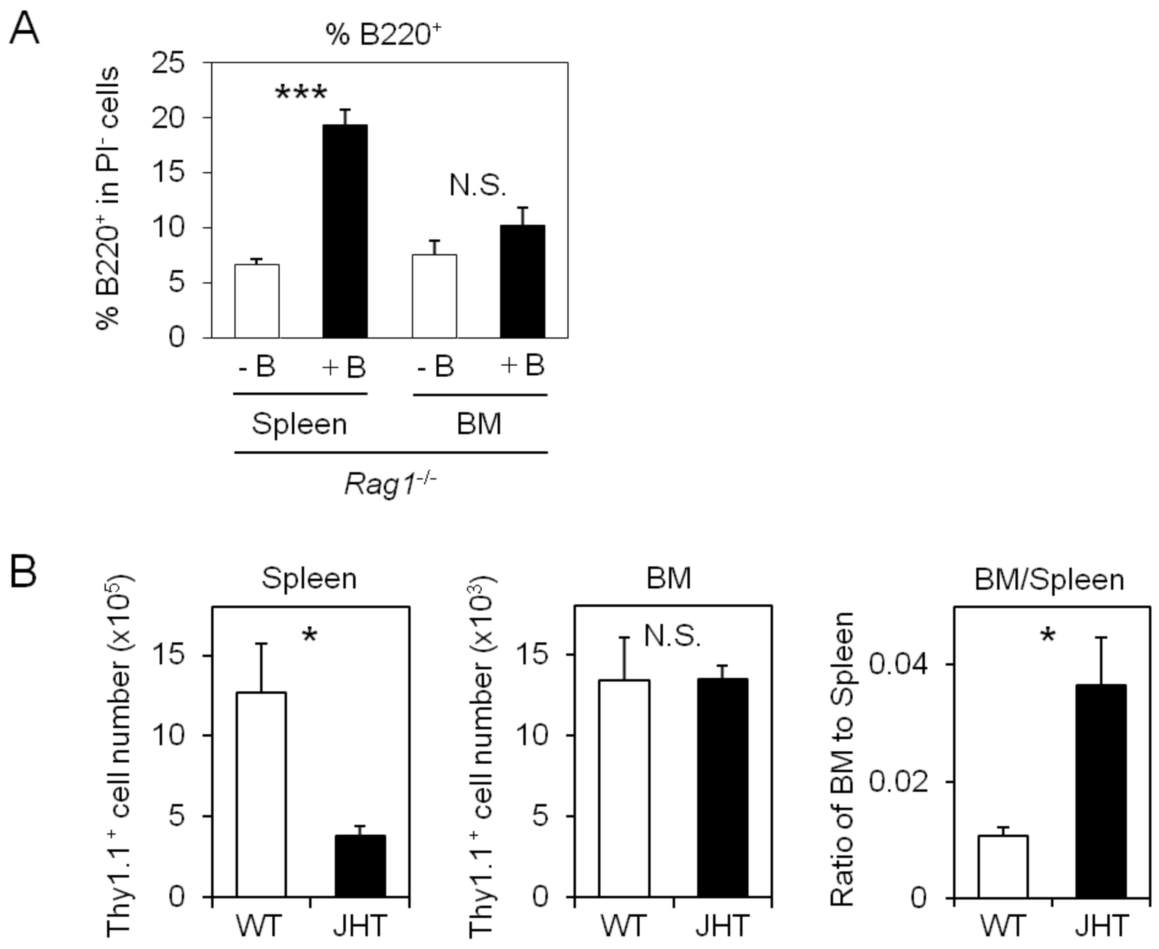

Figure S2 Accumulation of antigen-specific CD4 T cells in the BM is enhanced in B cell-deficient mice

A. Co-transferred B cells are detectable in the spleen but not BM of *Rag1*$^{-/-}$ mice. Purified Thy1.1^{+} LCMV-TCR CD4 T cells were transferred into *Rag1*-KO mice with or without B cells followed by immunization with LCMV $GP_{61\text{-}80}$ plus LPS. On day 6, the transferred B220^{+} cells in the spleen and BM were analyzed by flow cytometry. Bar chart represents the cell numbers. Data represent the mean ± SD. *** $p < 0.001$. N=4.

B. Accumulation of antigen-specific CD4 T cells in the BM is enhanced in B cell-deficient JHT host mice. Purified Thy1.1^{+} LCMV-TCR CD4 T cells were transferred into C57BL/6 or JHT mice followed by immunization with LCMV $GP_{61\text{-}80}$ plus LPS. On day 6, Thy1.1^{+} CD4 T cells in the spleen and BM were analyzed by flow cytometry and enumerated. Bar charts represent the cell numbers and migratory ratio from the spleen to the BM. Data represent the mean ± SD. * $p < 0.05$. N=3.

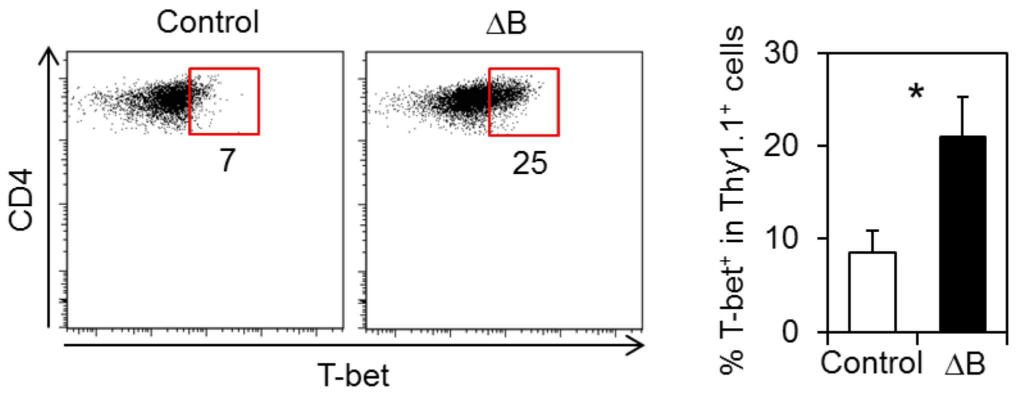

Figure S3 B cell-depletion increases T-bet$^+$ antigen-specific CD4 T cell population.
C57BL/6 mice were treated with isotype-matched control or anti-IgD antibody followed by injection of anti-rat IgG antibody. Two days later, purified Thy1.1$^+$ LCMV-TCR CD4 T cells were transferred into the antibody-treated mice, and then immunized with LCMV GP$_{61-80}$ plus LPS. On day 6, intracellular staining for T-bet was performed and a T-bet$^+$ population was analyzed by flow cytometry. Gating plots and bar chart show the T-bet$^+$ population in Thy1.1$^+$ CD4 T cells. Data represent the mean ± SD. * $p < 0.05$. N=3.

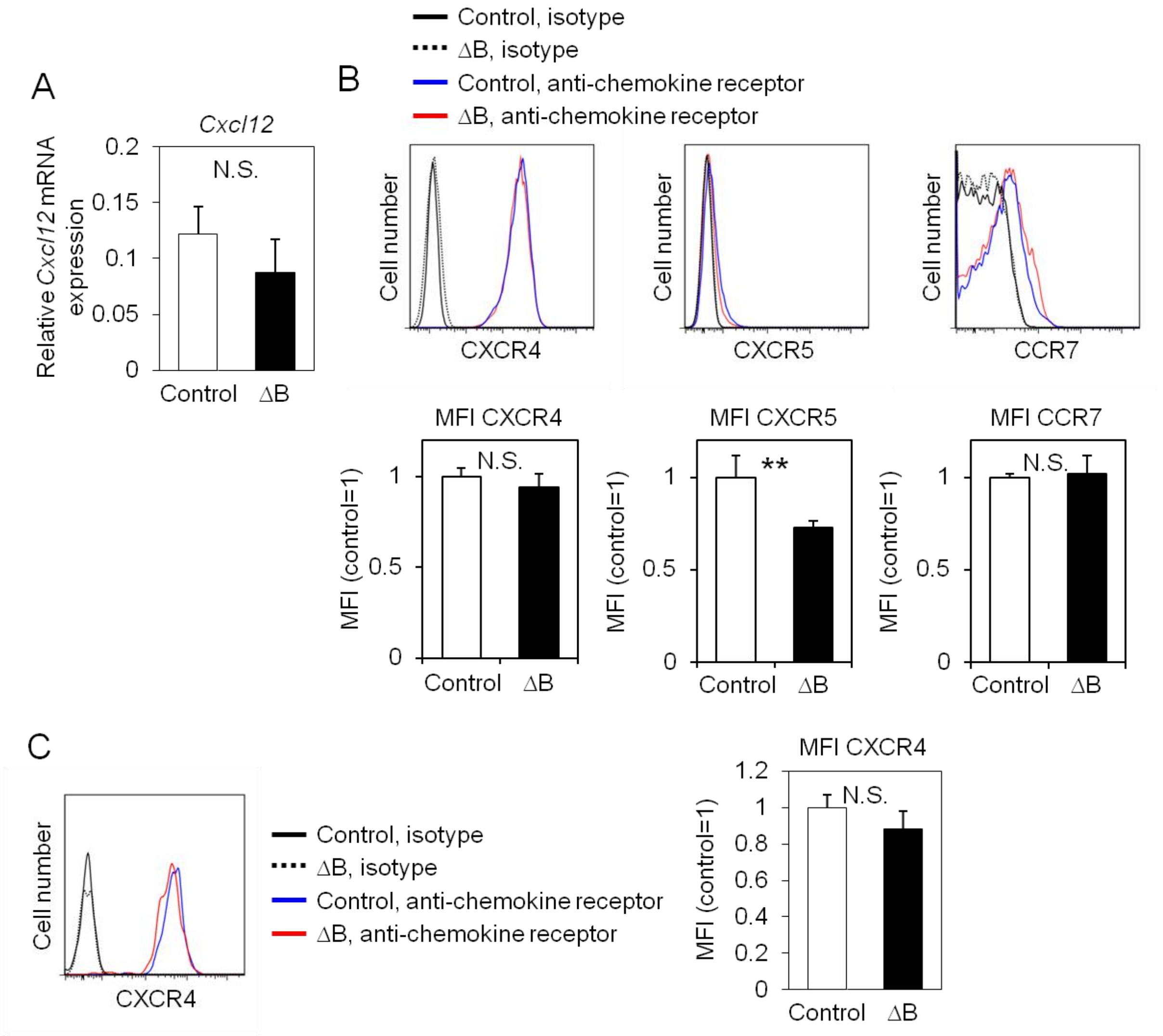

Figure S4 Effect of B cell depletion on the expression of chemokines and their receptors in the spleen

A. *Cxcl12* mRNA expression in the spleen is not affected by B cell depletion. The expression of *Cxcl12* mRNA was analyzed by real-time PCR analysis. Bar chart represents relative expression level of *Cxcl12* mRNA in the spleen. Data represents the mean ± SD. N=3-5.

B. The expression levels of CXCR4, CXCR5 and CCR7 in splenic antigen-specific activated CD4 T cells are not affected by B cell depletion. Purified Thy1.1$^+$ LCMV-TCR CD4 T cells were transferred into B cell-depleted or control C57BL/6 mice followed by immunization with LCMV GP_{61-80} plus LPS. On day 6, the indicated chemokine receptor expression was analyzed by flow cytometry. Histograms show chemokine receptor expression in CD4$^+$Thy1.1$^+$B220$^-$NK1.1$^-$PI$^-$ cells in the spleen. Bar charts represent the relative ratio of mean fluorescent intensity (MFI) of chemokine receptors. Data are representative of two independent experiments and represent the mean ± SD. ** $p < 0.01$. N=6.

C. The expression levels of CXCR4 in the BM-resident antigen-specific activated CD4 T cells are not affected by B cell depletion. As described in Fig. S4B, CXCR4 expression in the BM antigen-specific CD4 T cells was analyzed by flow cytometry. CXCR5 and CCR7 were not expressed on both BM CD4 T cells from B-cell depleted and control mice (data not shown). Histograms show CXCR4 expression in CD4$^+$Thy1.1$^+$B220$^-$NK1.1$^-$PI$^-$ cells in the BM. Bar chart represents the relative ratio of mean fluorescent intensity (MFI) of CXCR4. Data are representative of two independent experiments and represent the mean ± SD.

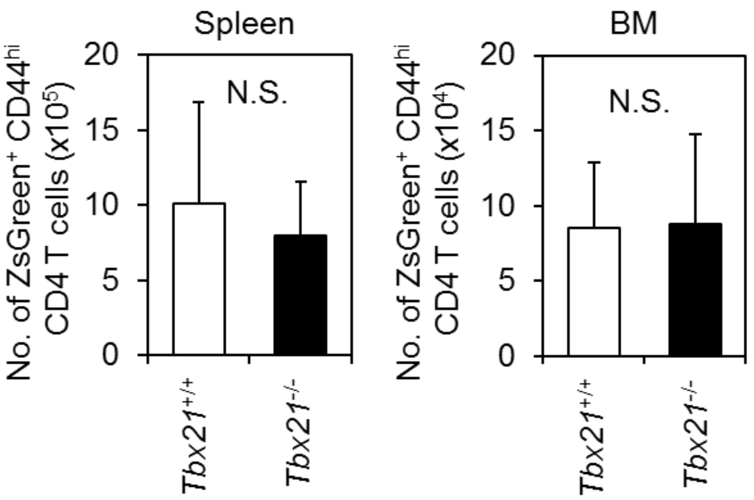

<u>Figure S5 Memory CD4 T cells reside in the spleen and BM independent on T-bet expression</u>
Memory CD4 T cells in the spleen and BM from T-bet-ZsGreen reporter mice in *Tbx21*$^{+/+}$ or *Tbx21*$^{-/-}$ background in steady state. Bar charts represent the numbers of ZsGreen^{+}CD3^{+}CD4^{+}CD44^{hi} cells in the spleen and BM. Data represent the mean ± SD. N=4.
